# Profile of Urological Emergency Admissions at a University Hospital in Morocco: A Retrospective Study Over 18 Months

**DOI:** 10.7759/cureus.118033

**Published:** 2026-10-16

**Authors:** Charaf Eddine Harchaoui, Brahim Masaoudi, Mohamed Amine Harchaoui, Mohamed Tetou, Mohammed Mrabti, Youness Boukhlifi, Abdessamad El Bahri, Mohamed Alami, Ahmed Ameur

**Affiliations:** 1 Urology, Mohammed V Military Hospital, Rabat, MAR

**Keywords:** acute renal colic, acute scrotum, epidemiology, gross hematuria, morocco, urological emergencies

## Abstract

Introduction: Urological emergencies represent a substantial component of hospital activity but remain poorly documented in Morocco. The aim of this study was to describe the epidemiological, clinical, and therapeutic profile of urological emergencies requiring hospitalization at a university hospital in Morocco, in order to establish a reliable reference database.

Methods: We conducted a retrospective descriptive study between January 2024 and June 2025 including all patients admitted for urological emergencies requiring hospitalization. Data were extracted from the hospital information system (HIS). Demographic characteristics, reasons for admission, complementary examinations, final diagnoses, treatments, and lengths of stay were analyzed.

Results: Among 2,106 hospitalizations, 339 were related to a urological emergency (16.09%). The population was predominantly male (79.6%; male-to-female ratio 3.9:1), with a mean age of 58.2 years and a peak incidence between 65 and 69 years. The most frequent emergencies were complicated renal colic (42.18%; 143 cases), macroscopic hematuria (17.40%; 59 cases), and acute scrotum (16.22%; 55 cases). Infectious emergencies accounted for 46% of admissions (156 cases), mainly obstructive pyelonephritis (57.05%) and complicated prostatitis (16.7%). The median length of hospital stay was three days, with longer stays observed in severe conditions such as Fournier’s gangrene and urinary peritonitis (up to seven days). A total of 241 patients (71.09%) required surgical intervention, primarily upper urinary tract drainage (61.8%) and exploratory scrototomy (22.4%).

Conclusion: In our setting, urological emergencies requiring hospitalization were dominated by complicated renal colic. The implementation of HIS and the involvement of multiple physicians have significantly improved patient traceability and represent a valuable tool for epidemiological analysis.

## Introduction

Urological emergencies are a frequent cause of medical consultation and require rapid management to prevent functional impairment and potentially life-threatening complications. Their prevalence and clinical presentation vary according to healthcare settings and organizational practices [1].

In Morocco, few studies have systematically assessed urological emergency activity within a university hospital [2,3]. The study center is a major referral hospital that manages emergencies among military personnel and their families and receives referrals from several regional military hospitals, as well as civilian patients, representing a catchment area of several hundred thousand inhabitants.

In Africa, and particularly in Senegal, a 20-month study involving 1,237 patients reported that 372 patients (30.1%) required emergency hospitalization, with 331 surgical procedures performed [4].

In France, a study reported that among 1,257 patients presenting to a urological emergency unit, approximately 45% required hospitalization in the urology department [5].

The management of urological emergencies relies on a multidisciplinary team. Initial evaluation and triage are carried out by the emergency medicine intern, while the nurse, assisted by a nursing aide, performs basic procedures such as catheterization, clot evacuation, and wound care. The on-call urology team includes a senior urologist and two residents, with a dedicated phone line allowing immediate contact with the urology resident on duty at any time.

The technical facilities include a consultation room equipped with essential emergency tools and an ultrasound machine. A nearby operating room enables prompt surgical management when needed, supported by an on-call operating room nurse and anesthesiologist.

This study aimed to evaluate the contribution of urological emergencies to the activity of the urology department at a university hospital in Morocco, describe the epidemiological characteristics of patients admitted for urological emergencies, and establish a reference database on the patterns of urological emergencies in our setting.

## Materials and methods

To achieve this objective, we used a hospital information system (HIS) widely implemented across healthcare institutions. This system enables centralized digital management of the entire patient care pathway from admission to discharge. It integrates computerized urological records that include the reason for consultation, clinical symptoms, diagnoses, prescriptions, test results, procedures performed, and operative reports.

We conducted a retrospective descriptive study involving 339 patients over an 18-month period, from January 2024 to June 2025. The study included all urological emergencies requiring initial hospitalization in the emergency department of the Mohammed V Military Teaching Hospital, Rabat, Morocco, followed by transfer either directly to the urology department for specialized management or via the emergency operating room.

Urological emergencies that did not require hospitalization (e.g., uncomplicated acute urinary retention, simple renal colic, uncomplicated prostatitis) were excluded.

Demographic characteristics included age, sex, and urological history. We analyzed the reasons for emergency admission, including complicated renal colic (pyelonephritis, acute renal failure, or severe pain), macroscopic hematuria, acute scrotum, complicated prostatitis (sepsis, abscess, or urinary retention), acute urinary retention complicated by renal failure, urogenital trauma, Fournier’s gangrene of urological origin, and urinary peritonitis.

All complementary examinations performed during hospitalization were collected and analyzed: biological tests (urine cytobacteriological examination and associated laboratory results) and imaging studies (plain abdominal radiography, ultrasound, CT scan). Final diagnoses and therapeutic interventions were recorded. Length of hospital stay and procedures performed during admission were also documented.

A standardized data extraction form was designed using Google Forms (Google LLC, Mountain View, USA) to ensure consistent data collection from the HIS. The data were entered into Microsoft Excel (Microsoft Corp., Redmond, USA) for initial organization and subsequently exported to Jamovi version 2.6.44 (The Jamovi Project; https://www.jamovi.org) for statistical analysis. The results were summarized as simple frequency tables and percentages.

Finally, it should be noted that due to incomplete records in the HIS, it was not possible to assess the total number of urological emergencies relative to other emergency cases, nor to capture urological emergencies that did not require hospitalization.

The study was conducted in accordance with the principles of the Declaration of Helsinki.

## Results

Over the 18-month study period, urological emergencies requiring hospitalization accounted for 16.09% of all admissions to the urology department, corresponding to 339 patients out of a total of 2,106. There was a marked male predominance, with 270 men (79.6%) compared to 69 women (20.4%), yielding a male-to-female ratio of 3.9:1. The mean patient age was 58.2 years, with extremes ranging from 14 to 98 years. A unimodal age peak was observed in the 65-69-year age group, which included 55 patients (16.22%). Only one patient (0.29%) was under the age of 15, and three patients (0.88%) were over 95 years old, as illustrated in Table 1.

**Table 1:** Age distribution showing a unimodal peak (n=339).

Age group (years)	n	%
10-15	1	0.29
15-20	24	7.08
20-25	15	4.42
25-30	10	2.95
30-35	12	3.54
35-40	10	2.95
40-45	14	4.13
45-50	7	2.06
50-55	21	6.19
55-60	30	8.85
60-65	30	8.85
65-70	55	16.22
70-75	37	10.91
75-80	24	7.08
80-85	22	6.49
85-90	19	5.6
90-95	5	1.47
95-100	3	0.88
Total	339	100

The most frequent urological emergencies differed slightly between men and women. Among men, the leading causes were complicated renal colic, acute scrotum, and macroscopic hematuria. Among women, the most common were complicated renal colic, macroscopic hematuria, and catheter dislodgement. Complicated renal colic was the most frequent emergency, with 143 cases recorded over the 18-month period, corresponding to an average of approximately 95 cases per year. Between January 2024 and June 2025, the main urological emergencies requiring hospitalization were distributed as follows: complicated renal colic (hyperalgesic, obstructive pyelonephritis, or associated with acute renal failure): 42.18% (143/339); macroscopic hematuria: 17.40% (59/339), acute scrotum: 16.22% (55/339); complicated prostatitis: 7.67% (26/339); catheter dislodgement (nephrostomy or single-J): 4.72% (16/339); urological trauma (retroperitoneal, pelvic, or external genital organs): 4.13% (14/339); abscesses (psoas, perineal, or inguinal): 2.65% (9/339); Fournier’s gangrene: 2.06% (7/339); acute urinary retention with associated acute renal failure: 2.06% (7/339); and urinary peritonitis: 0.88% (3/339) (Table 2).

**Table 2:** Cause of admission, median age, and median length of hospitalization according to the cause of admission (n=339).

Cause of admission	Gender						Median length of hospitalization (days)	Median age (years)
	Female		Male		Total			
	n	%	n	%	n	%		
Complicated renal colic	53	76.81	90	33.33	143	42.18	4	60
Macroscopic hematuria	7	10.14	52	19.26	59	17.40	6	74
Acute scrotum	0	0.00	55	20.37	55	16.22	1	21
Complicated prostatitis	0	0.00	26	9.63	26	7.67	6	70.5
Catheter falls (nephrostomy, single-J)	5	7.25	11	4.07	16	4.72	2	72
Urogenital trauma	0	0.00	14	5.19	14	4.13	2.5	31.5
Abscesses (psoas, perineal, inguinal)	2	2.90	7	2.59	9	2.65	4	56
Fournier's gangrene	0	0.00	7	2.59	7	2.06	7	69
Acute urinary retention complicated by acute renal failure	2	2.90	5	1.85	7	2.06	3	74
Urinary peritonitis	0	0.00	3	1.11	3	0.88	7	56
Total	69	100.00	270	100.00	339	100.00	3	64

Among the 339 urological emergencies requiring hospitalization over the 18-month period - representing an average of 226 cases per year - 110 patients underwent a technical procedure performed under local anesthesia, mainly bladder catheterization or suprapubic catheter placement. In contrast, 241 admissions required management in the emergency operating room. The most frequently performed procedures were upper urinary tract drainage (double-J stent, nephrostomy, or single-J stent): 149 cases (61.83%); exploratory scrototomy: 54 cases (22.41%); and electrocoagulation for macroscopic hematuria: 10 cases (4.15%) (Table 3).

**Table 3:** Procedures performed in the emergency operating room (n=241).

Procedure	n	%
Drainage of the upper urinary tract (double-J stent, nephrostomy, or single-J stent)	149	61.83
Exploratory scrototomy	54	22.41
Electrocoagulation	10	4.15
Necrosectomy	7	2.90
Surgical drainage (abscess)	7	2.90
Surgical repair of penile fracture	7	2.90
Others (nephrectomy, cystectomy, lumbotomy, laparotomy)	7	2.90
Total	241	100.00

The median length of hospital stay was three days. Patients with Fournier’s gangrene or urinary peritonitis had longer stays, with a median of seven days, whereas those with acute scrotum required only one day on average (Table 2). Infectious emergencies accounted for 156 of the 339 cases, representing 46.02% of all hospitalizations. The majority of these patients received antibiotic therapy, primarily a combination of ceftriaxone and gentamicin. The three most frequent infectious emergencies were obstructive pyelonephritis (89 cases, 57.05%), complicated prostatitis (26 cases, 16.67%), and scrotal abscesses and orchiepididymitis (21 cases, 13.46%) (Table 4).

**Table 4:** Infectious emergencies (n=156).

Cause of admission	n	%
Obstructive pyelonephritis	89	57.05
Prostatitis	26	16.67
Orchitis-epididymitis, scrotal abscess	21	13.46
Abscess (psoas, perineal, inguinal)	9	5.77
Fournier's gangrene	7	4.49
Urinary peritonitis	3	1.92
Other (emphysematous cystitis)	1	0.64
Total	156	100.00

Radiological examinations (ultrasound and/or computed tomography) were performed in 334 patients (98.52%).

Complicated renal colic

Complicated renal colic was the most frequent cause of urological emergencies requiring hospitalization in both sexes. Over the 18-month study period, the HIS recorded 143 hospitalizations for complicated renal colic, representing 42.18% of all emergencies requiring admission. Among these were obstructive pyelonephritis (isolated and associated with renal failure): 89 patients (62.24%); obstructive renal failure (isolated and associated with pyelonephritis): 52 patients (36.35%); and hyperalgesic renal colic: 27 patients (18.88%). The total number of individual complications exceeded the total number of complicated renal colic cases (n=143) (Table 5). Men accounted for 62.94% (90/143) of cases. The median age was 60 years, and the median length of hospital stay was four days (Table 2). Emergency drainage was performed in the majority of patients using a double-J stent or nephrostomy.

**Table 5:** Complicated renal colic (n=143).

Complicated renal colic	n	%
Isolated pyelonephritis	64	44.76
Isolated acute kidney failure	27	18.88
Pyelonephritis associated with acute renal failure	25	17.48
Hyperalgesic	27	18.88
Total	143	100.00

Macroscopic hematuria

The hospitalization rate for macroscopic hematuria during the 18-month study period was 17.40% (59 out of 339 cases) (Table 2). The main causes were bladder tumors (45.76%, 27/59), followed by post-radiation cystitis and bedsores, each observed in 15.25% of cases (9/59) (Table 6). The most frequently performed hemostatic procedure was simple clot evacuation combined with continuous bladder irrigation, carried out in 52 patients (88.14%). Endoscopic electrocoagulation was required in 10 patients (16.95%), while two patients underwent cystectomy. Blood transfusions were necessary in 54.24% of patients (32/59) (Table 7). The median age of patients was 74 years, and the median length of hospital stay was six days (Table 2).

**Table 6:** Causes of macroscopic hematuria (n=59). TURP: transurethral resection of the prostate; TURBT: transurethral resection of bladder tumor

Causes of macroscopic hematuria	n	%
Bladder tumor	27	45.76
Post-radiation cystitis	9	15.25
Scab falls (post-TURP/TURBT)	9	15.25
Benign prostatic hyperplasia	6	10.17
Prostate adenocarcinoma	3	5.08
Unknown	3	5.08
Tumor of the upper urinary tract	1	1.69
Kidney tumor	1	1.69
Total	59	100.00

**Table 7:** Emergency hemostasis procedures and transfusion use (n=59).

Hemostasis procedures/transfusion	n	%
Bladder washout	52	88.14
Electrocoagulation	10	16.95
Washout under cystoscopy	7	11.86
Cystectomy	2	3.39
Transfusion	32	54.24
Total	59	100.00

Acute scrotum

The majority of patients hospitalized for acute scrotum (92.73%, 51 out of 55 patients) underwent exploratory scrototomy. The main causes were testicular torsion (67.27%, 37/55 cases) and orchiepididymitis (20%, 11/55 cases). Additionally, seven patients presented with a testicular abscess (12.73%). The median age of patients with acute scrotum was 21 years (Table 2).

Urogenital trauma

Urogenital trauma accounted for 14 out of 339 cases (4.13%), with a male predominance of 100% (Table 2). Trauma to the external genital organs was the most frequent, including penile fractures (50%, 7/14) and scrotal injuries (28.57%, 4/14), all of which underwent surgical exploration. Additionally, three renal injuries were diagnosed: only one patient required a hemostatic nephrectomy, while the other two were managed conservatively with clinical and radiological monitoring.

Complicated prostatitis

Complicated prostatitis was the fourth most frequent cause of hospitalization, accounting for 26 out of 339 cases (7.67%) (Table 2). Among these patients, two cases were iatrogenic (7.7%), occurring after transurethral resection of the prostate. Nineteen patients had known benign prostatic hyperplasia (73.08%), and one patient had a urethral stricture (3.85%). The most frequently identified pathogen in urine cultures was *Escherichia coli *(5/13 positive cultures), followed by *Klebsiella pneumoniae* (3/13) and *Streptococcus agalactiae* (2/13). Complications were dominated by sepsis (11/26), followed by acute urinary retention and prostatic abscess, each observed in three out of 26 cases.

Catheter dislodgement

Catheter dislodgement (nephrostomy, single-J stent) accounted for 4.72% of hospitalizations (16/339) (Table 2), predominantly involving nephrostomy tubes (15 out of 16 cases), with a single case of single-J catheter dislodgement in a patient who had previously undergone cystectomy with ureterostomy. For 15 of the 16 patients, admission to the emergency department was required for catheter reinsertion.

Acute urinary retention complicated by renal failure

Over the 18-month study period, the department recorded seven cases of acute urinary retention complicated by acute renal failure, representing 2.06% of hospital admissions (7/339). The median age of these patients was 74 years, the median length of hospital stay was three days, and 71.43% were men (Table 2).

Fournier's gangrene

Over the 18-month period, seven cases of urinary Fournier's gangrene were hospitalized in the department, all of whom were male patients. The length of hospitalization ranged from one to 75 days, with a median of seven days (Table 2). Among them, three patients were known to have urethral stenosis. Six patients received triple antibiotic therapy: ceftriaxone + gentamicin + metronidazole for five patients, piperacillin-tazobactam + gentamicin + metronidazole for one patient, and one patient was discharged the next day on protected amoxicillin.

Urinary peritonitis

Among the three cases of urinary peritonitis, two were iatrogenic, occurring after radical prostatectomy and partial nephrectomy, respectively. The third case resulted from spontaneous bladder rupture in a patient with a hypertonic bladder. All patients underwent exploratory laparotomy, and the median length of hospital stay was seven days (Table 2).

## Discussion

To our knowledge, this study is one of the first to systematically describe urological emergency admissions at a Moroccan university hospital. In recent months, the continuous improvement of the HIS, combined with the active involvement of medical teams, has significantly enhanced the quality of hospital data, with a direct impact on both clinical research and patient care.

Urological emergencies represent an important component of our department’s activity, with an average of 18.83 hospitalizations per month. This figure is considerably higher than that reported in Senegal, where Diabaté et al. [6] described an average of three cases per month and Diallo et al. [7] reported 5.45 cases per month-differences often attributed to delayed consultation and reliance on traditional medicine, which reduce hospitalization rates. In contrast, our results are comparable to those observed in France by Boissier et al. [8], who recorded an average of 19.6 urological emergencies per month per department in 2019.

In our study, the most common urological emergencies requiring hospitalization were complicated renal colic, macroscopic hematuria, and acute scrotum. This distribution aligns with French data, where the emergencies most frequently leading to hospitalization include obstructive renal failure, torsion of the spermatic cord, trauma to the pelvis and external genitalia, and macroscopic hematuria [8]. Conversely, in African contexts - particularly in Senegal - the leading urological emergencies necessitating immediate hospital admission remain acute scrotal swelling and Fournier’s gangrene [7].

The average age of patients was 58.2 years, confirming that urological emergencies predominantly affect an older population. As reported in most series, we also observed a marked male predominance [6-10]. Regarding urinary lithiasis, men typically have higher urinary osmolarity than women, which favors crystallization and stone formation [11]. For macroscopic hematuria - mainly linked in our study to bladder tumors - the predominance of male patients may be explained by the higher prevalence of smoking and occupational exposure to carcinogens in men, both well-established risk factors for bladder cancer [12].

Similar to European data, renal colic is the leading cause of urological hospitalization. In France, it accounts for approximately 1-2% of all annual emergency room visits, with 23% requiring admission [5]. This high frequency is attributed to the steady increase in lithiasis observed in Western countries, driven by risk factors such as obesity, sedentary lifestyle, hyperuricemia, hypertension, diabetes, and dietary habits. In our context, lifestyle changes are gradually aligning the prevalence of urinary lithiasis with that observed in Western populations, currently affecting nearly 10% of the general population [5,13].

Consistent with our findings, European data show a high frequency of macroscopic hematuria in emergency settings. In France, Boissier et al. [8] report that macroscopic hematuria is one of the most common reasons for consultation after renal colic and acute urinary retention, with almost 44% of cases requiring hospitalization. Furthermore, as in our study, the diagnostic yield for malignancy is significantly higher among patients presenting with macroscopic hematuria, predominantly due to bladder tumors, a condition that mainly affects older adults [14]. In West Africa, the trends are similar, although the underlying etiologies differ: in Senegal, Diallo et al. [7] identified macroscopic hematuria as the fourth leading cause of emergency admission, often associated with severe and advanced diseases, particularly bladder cancer linked to schistosomiasis in endemic regions.

Although acute scrotum is a relatively uncommon condition, it frequently necessitates surgical exploration once ultrasound findings suggest spermatic cord torsion. In Senegal, as in our series, it is the second most common urological surgical emergency [7]. In France, it ranks ninth among the most frequent urological emergencies in men, with a hospitalization rate of 80% [8].

Similar to our results, trauma to the external genital organs represents the most common form of urogenital trauma reported in the literature, with a clear predominance of scrotal injuries [4,5,7].

Prostatitis was the fourth most common cause of hospitalization in our department. In France, it is the second most frequent infectious urological emergency requiring hospitalization, whereas in our context, it ranks third among infectious causes [8]. This condition often requires specialized care due to the risk of potentially serious or life-threatening complications [8]. In contrast, a study conducted in Senegal reported prostatitis as the least common infectious emergency, with only one case among 436 consultations [7].

Catheter dislodgement - particularly nephrostomy tube displacement - represents another significant reason for emergency room visits and frequently requires transfer to the operating room for repositioning. Several risk factors have been identified; notably, patients with renal parenchyma thickness below 16 mm and posterolateral muscular wall thickness below 8 mm are at higher risk [15]. Identifying these factors allows for enhanced patient monitoring and the adoption of preventive technical strategies [15]. 

Acute urinary retention is a common and serious urological emergency, ranking among the top three urological emergencies in men [8]. Without prompt and adequate management, it can progress to acute renal failure, making immediate hospitalization necessary in most cases [8]. This underscores the importance of early screening: even during non-urological consultations, male patients should be questioned about lower urinary tract symptoms (LUTS) - such as urinary discomfort, weak stream, pollakiuria, nocturia, or post-void residual sensation - to identify individuals at risk and initiate timely management [16].

Fournier’s gangrene is a severe medical and surgical emergency, particularly prominent in African series. It requires immediate surgical intervention - typically debridement of the external genital organs - sometimes combined with cystostomy for urinary diversion. Repeated surgical debridement may be necessary to remove all necrotic tissue and prevent further extension of the infection [4]. In African contexts, Fournier’s gangrene often arises as a complication of urethral stricture [7]. Fortunately, in our setting, this condition remains rare, with a frequency comparable to that reported in Western countries. A survey of European urologists found that urology departments treated an average of 4.2 ± 3.11 cases per year [17].

Urinary peritonitis is an extremely rare but severe condition requiring urgent diagnosis and management. Spontaneous ruptures are far less common than secondary ruptures. Diagnosis typically relies on delayed-phase imaging (urography), sometimes supplemented by cystography [18].

Our study has certain limitations, notably the absence of an assessment of the total number of visits to the emergency department for urological reasons, which limits the representativeness of all urological emergencies.

## Conclusions

Hospitalization-requiring urological emergencies constitute a significant part of our clinical activity. In our setting, the most common presentations were renal colic, macroscopic hematuria, and acute scrotum. These emergencies predominantly affected elderly men. Among infectious urological emergencies, acute pyelonephritis was the most common presentation. These findings underscore the importance of prompt diagnosis and appropriate management to reduce complications and preserve renal and functional outcomes.
